# Supplementary material for: The structure of phosphatidylinositol remodeling MBOAT7 reveals its catalytic mechanism and enables inhibitor identification
Source: Nat Commun. 2023 Jun 14;14:3533. doi: 10.1038/s41467-023-38932-5 (PMC10267149; doi:10.1038/s41467-023-38932-5)
Supplement: Supplementary file 1 — Supplementary Inforamtion File [file 41467_2023_38932_MOESM1_ESM.docx]

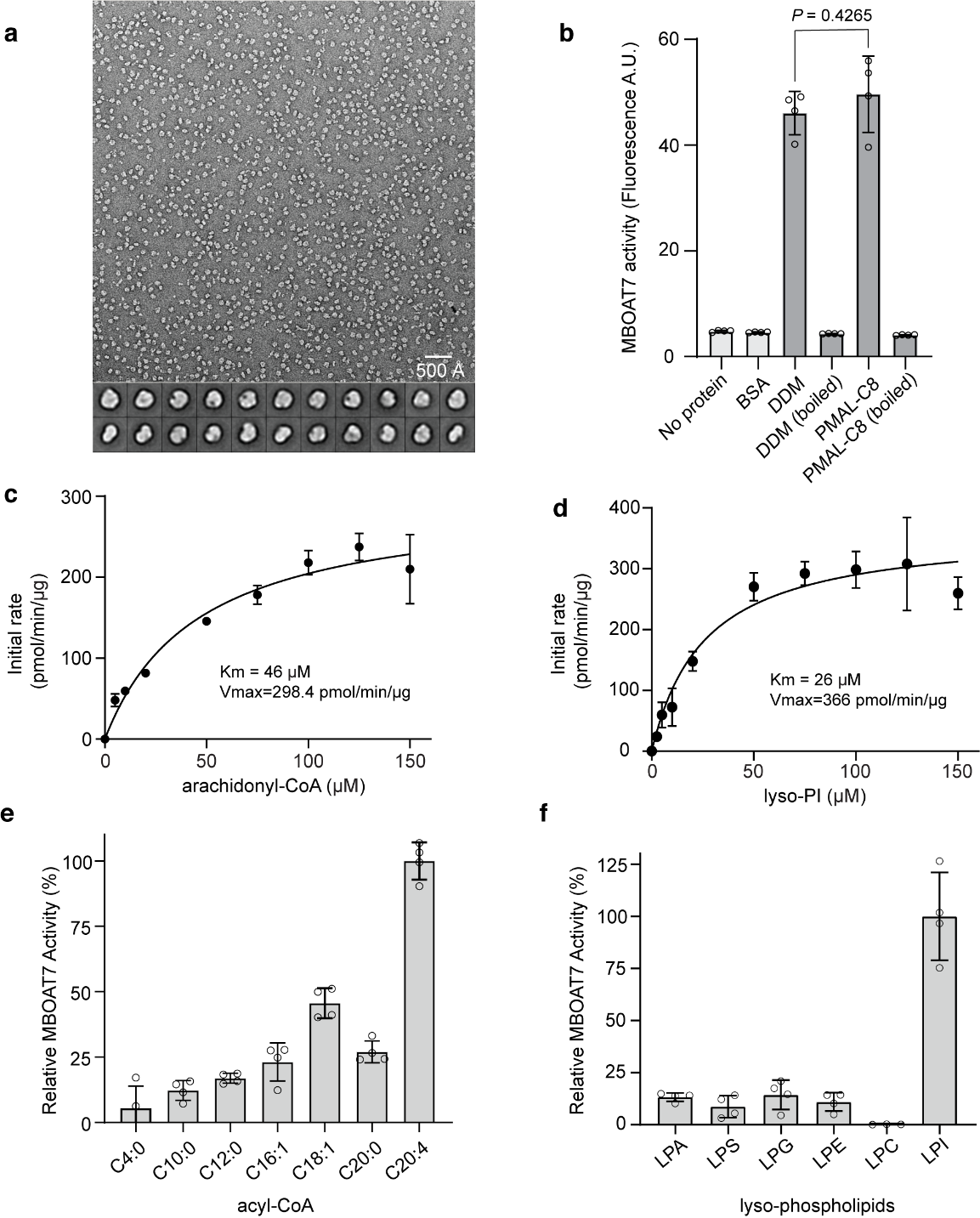


**Supplementary Fig. 1 | Characterization of purified human MBOAT7. a,** Representative negative-stain electron micrograph and 2D averages of purified MBOAT7 in PMAL-C8. Experiments were independently repeated 3 times with similar results. **b**, Activity comparison of purified MBOAT7 in DDM detergent or PMAL-C8. The activity is quantified by monitoring the fluorescent production of Co-SH that is covalently linked to CPM (mean ± SD, *n*=4 independent experiments). Analysis was performed using unpaired *t* test. **c** and **d**, Initial rate of reaction versus two substrates, arachidonyl-CoA (c) and lyso-PI (d) (mean ± SD, *n*=3 independent experiments). **e**, MBOAT7 activity towards acyl-CoA with different acyl chains (number of carbons : number of double bonds). mean ± SD, *n*=4 independent experiments. **f,** MBOAT7 activity towards different lyso-phospholipids. mean ± SD, *n*=4 independent experiments. LPA, lyso-phosphatidic acid; LPS, lyso-phosphatidylserine; LPG, lyso-phosphatidylglycerol; LPE, lyso-phosphatidylethanolamine; LPC, lyso-phosphatidylcholine; LPI, lyso-phosphatidylinositol.


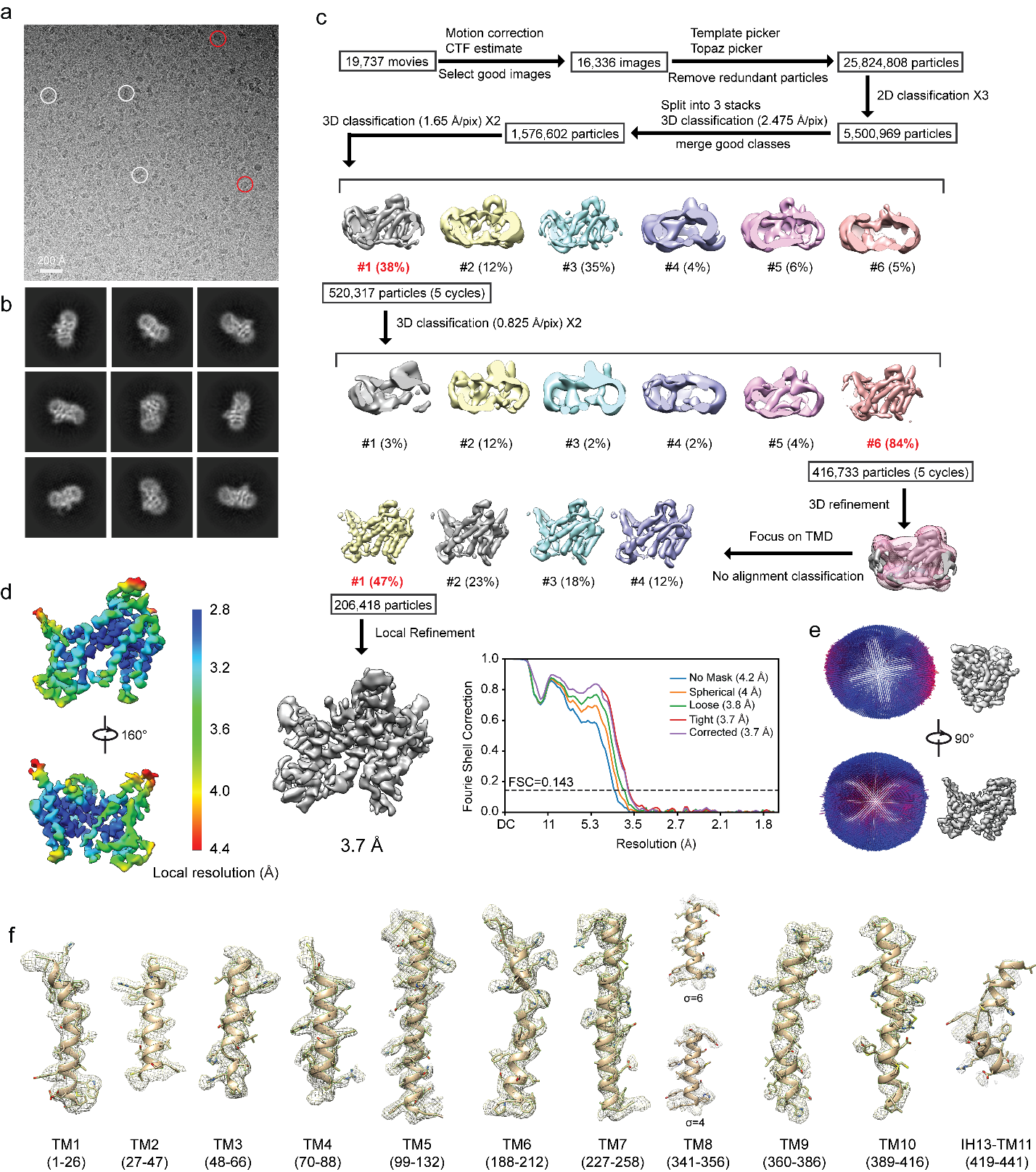


**Supplementary Fig. 2 | Cryo-EM image processing of human MBOAT7 in PMAL-C8. a,** Representative cryo-EM image of MBOAT7. Some particles are highlighted with white (side views) or red (end-on views) circles. **b**, Representative 2D class averages. **c**, A flow chart for data processing (see Methods for details) and its FSC curves between two half maps with indicated resolutions at FSC=0.143. **d**, Local-resolution map of MBOAT7 in two orientations. **e**, Angular distribution of particle images included in the final 3D reconstruction. **f,** Cryo-EM densities superimposed with atomic model for individual transmembrane helices (TM1-TM11). Maps are contoured at 6 σ, except TM8 which is shown at two contour levels.


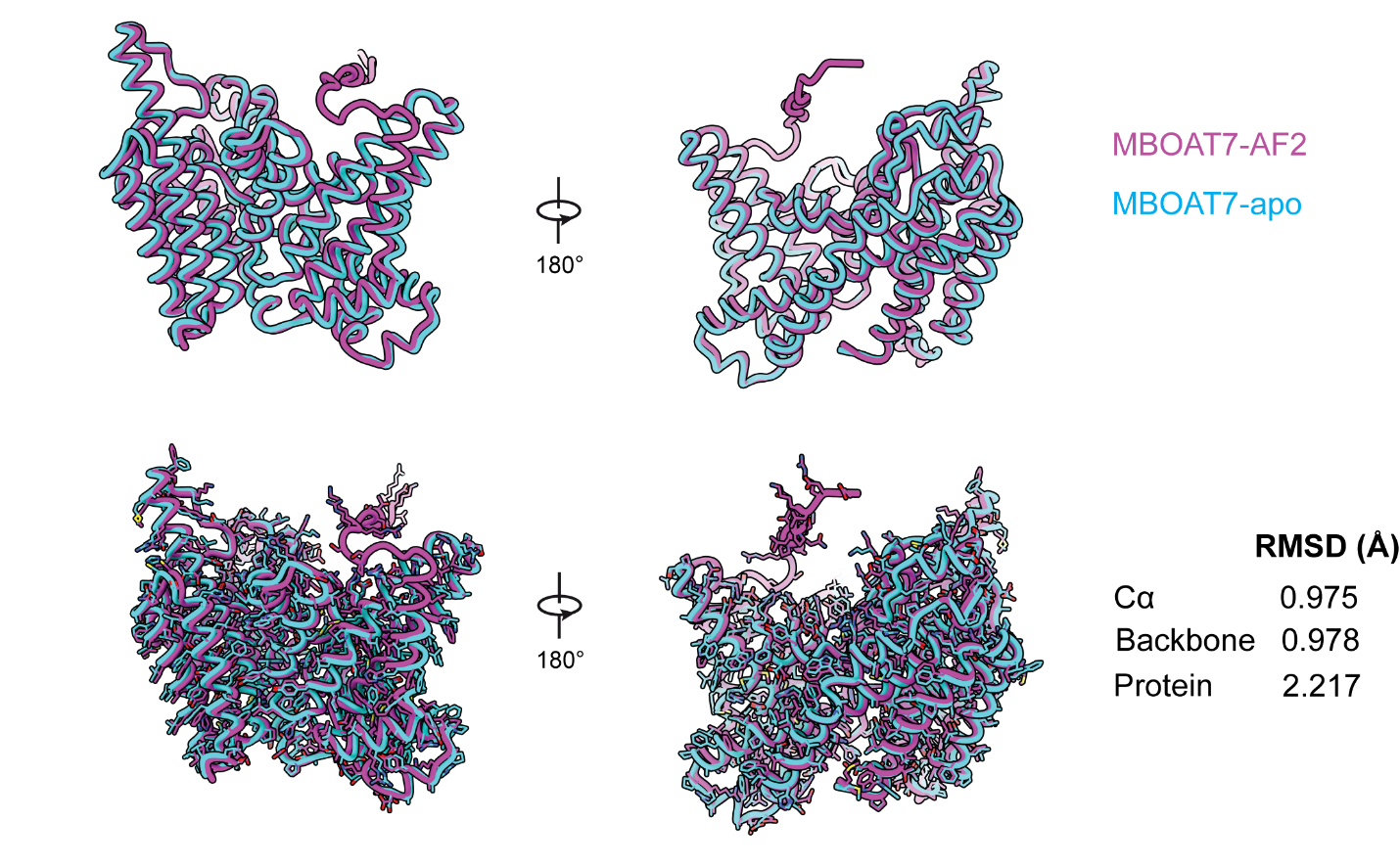


**Supplementary Fig. 3 | Comparison of the cryo-EM and AlphaFold2-predicted structures of MBOAT7.** Top, comparison of the backbones. Bottom, comparison of the entire proteins with side chains. The RMSD were calculated according to the cryo-EM solved 1–441 residues.


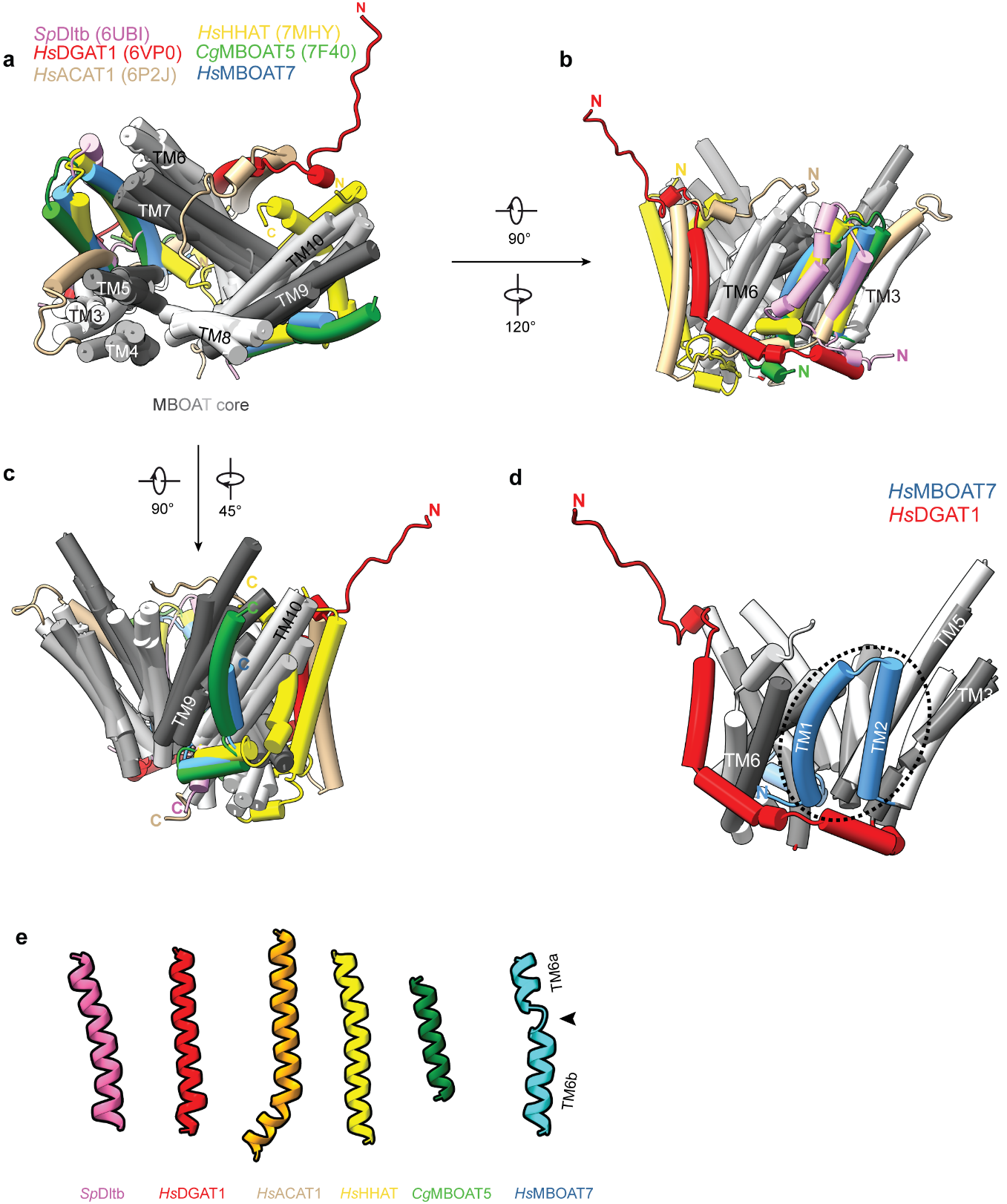


**Supplementary Fig. 4 | Comparison of MBOAT structures. a**, MBOAT structures are superimposed. The TMs of the MBOAT7 core are shown in different shades of grey, and the distinct N- and C-termini are colored so as to be consistent with the legend colors. **b** and **c**, Two different orientations to present the variable N (b) and C (c) terminal regions. **d**, Comparison between MBOAT7 and DGAT1. MBOAT7’s N terminal TM1-TM2 (cyan) blocks the lateral gate, and DGAT1’s N-terminus (red) is longer and involved in protein dimerization. **e**, Comparison of MBOAT7 TM6 to the corresponding TMs of other MBOAT proteins. A black arrow denotes the break of MBOAT7 TM6.


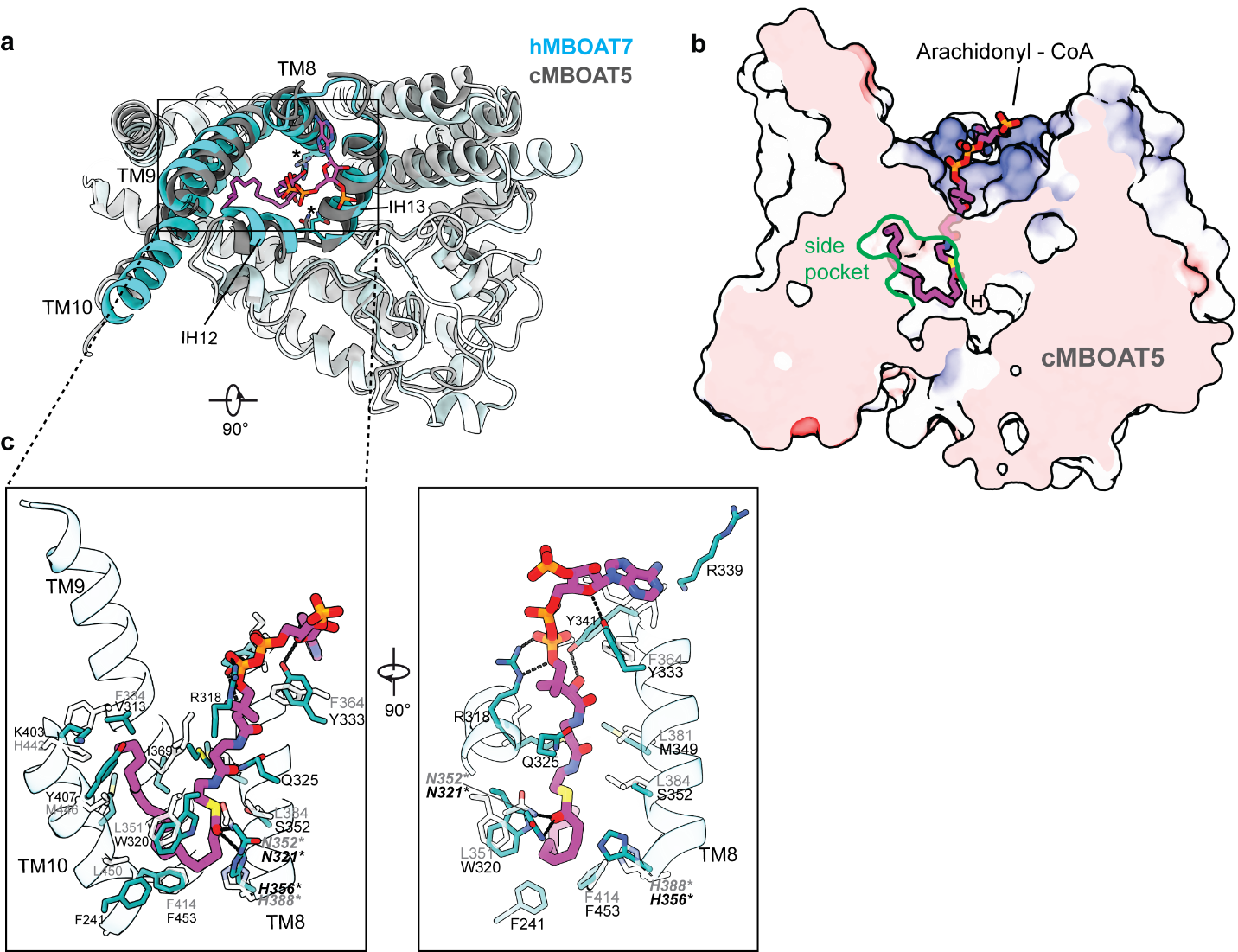


**Supplementary Fig. 5 | Comparison of the acyl-CoA access channels in human MBOAT7 and chicken MBOAT5. a**, Top view of the arachidonyl-CoA molecule docked into the MBOAT7 cytosolic channel. Human MBOAT7 (hMBOAT7) structure is superimposed with the chicken MBOAT5 structure complexed with arachidonyl-CoA (PDB 7F40). **b**, A cutting-in view of the cMBOAT5 catalytic chamber and side pocket bound with arachidonyl-CoA, for comparison with Fig. 2b. MBOAT5 is represented with electrostatic surfaces. H indicates the catalytic histidine. **c**, The interaction between arachidonyl-CoA and MBOAT7 residues. The view is the same to Fig. 2c except that the residues were labeled in pairs with the corresponding ones from cMBOAT5 (grey).


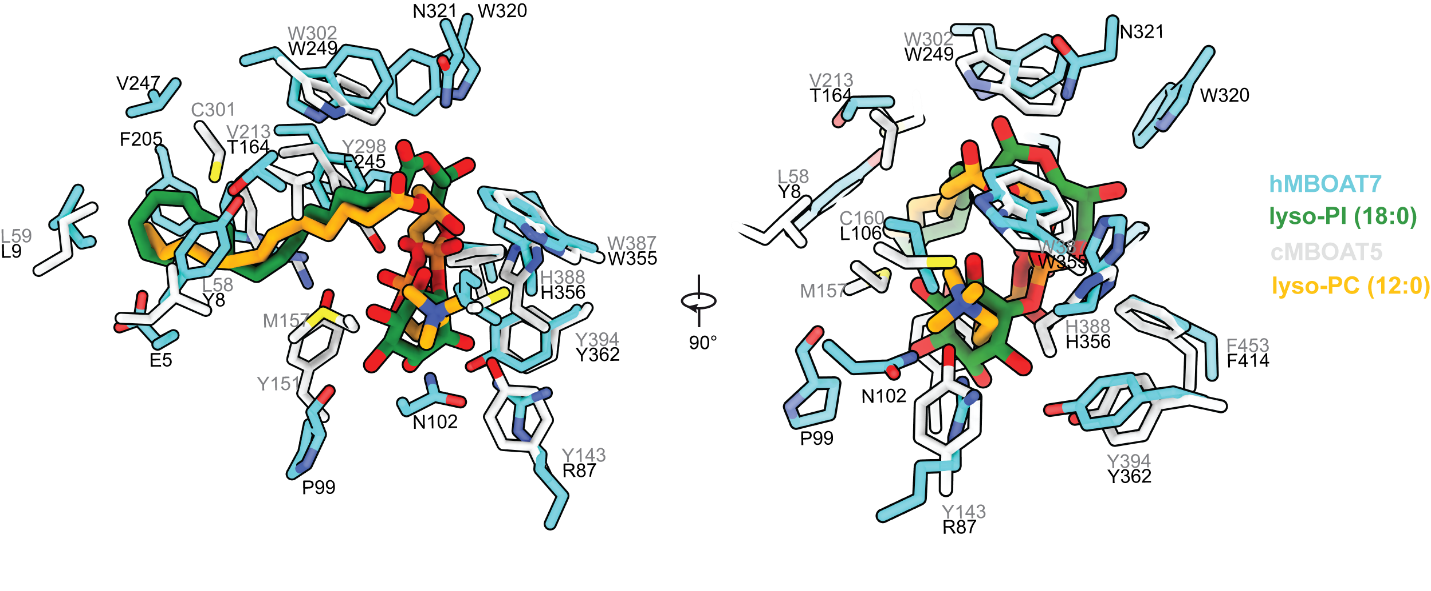


**Supplementary Fig. 6 | Comparison of the lyso-phospholipid interaction patterns in human MBOAT7 and chicken MBOAT5.** The cMBOAT5 with lyso-PC structure (PDB 7F3X) is superimposed with the energy-minimized hMBOAT7 structure bound with lyso-PI (same as in Fig. 3a–d). Only the lyso-phospholipid molecules and the key residues are shown.


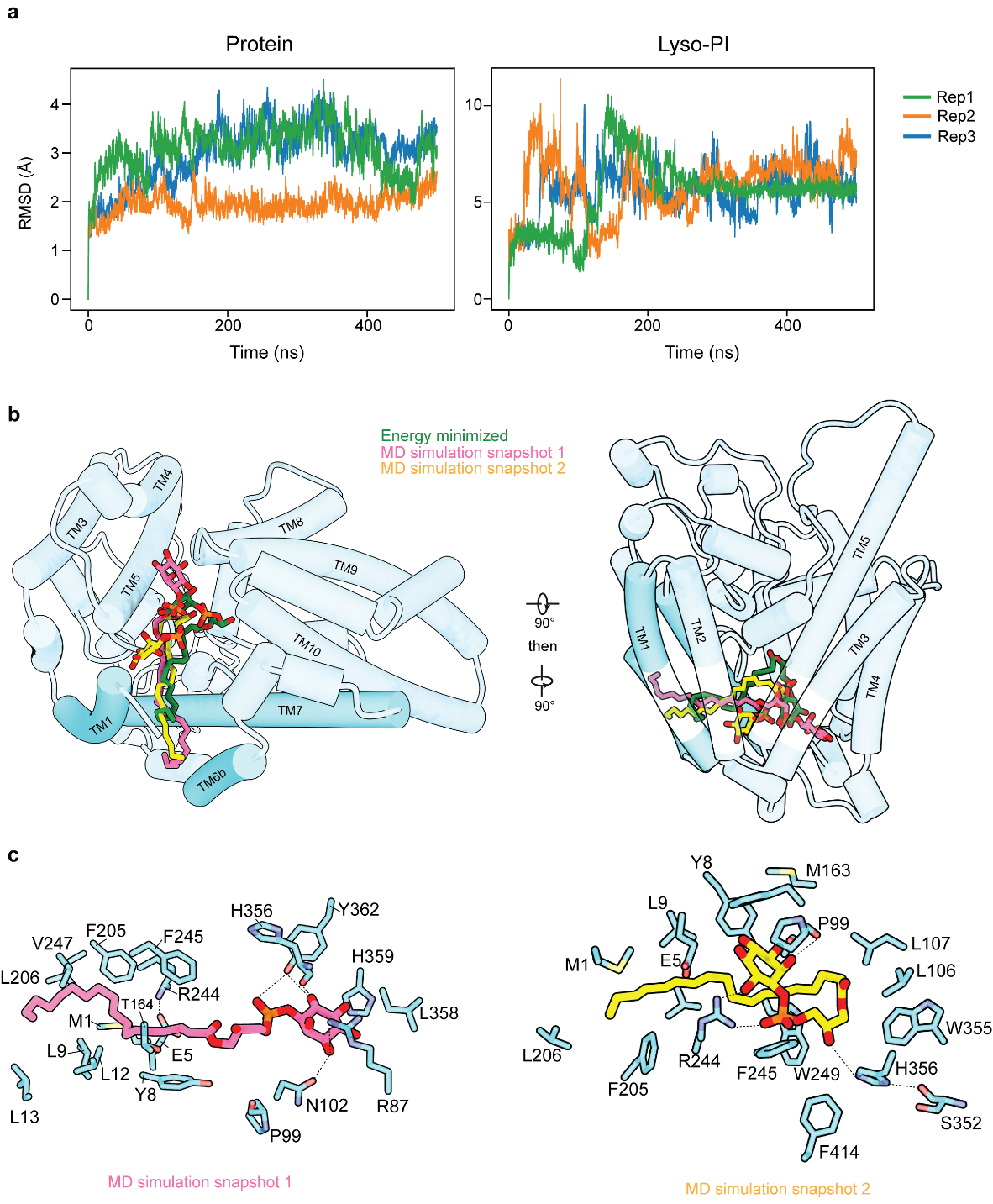


**Supplementary Fig. 7 | Analysis of molecular dynamics simulations of structure of MBOAT7 bound to lyso-PI. a**, Stability of the MBOAT7 protein and lyso-PI during MD simulations. Three 500-ns repeats were performed. RMSD of protein and lyso-PI with Cɑ atoms aligned against their position in the first frame. **b**, Comparison of lyso-PI conformations during MD simulations. Two transiently stable conformations most are shown to compare with the initial state. **c**, Detailed interactions between MBOAT7 and lyso-PI in the two transiently stable snapshots.


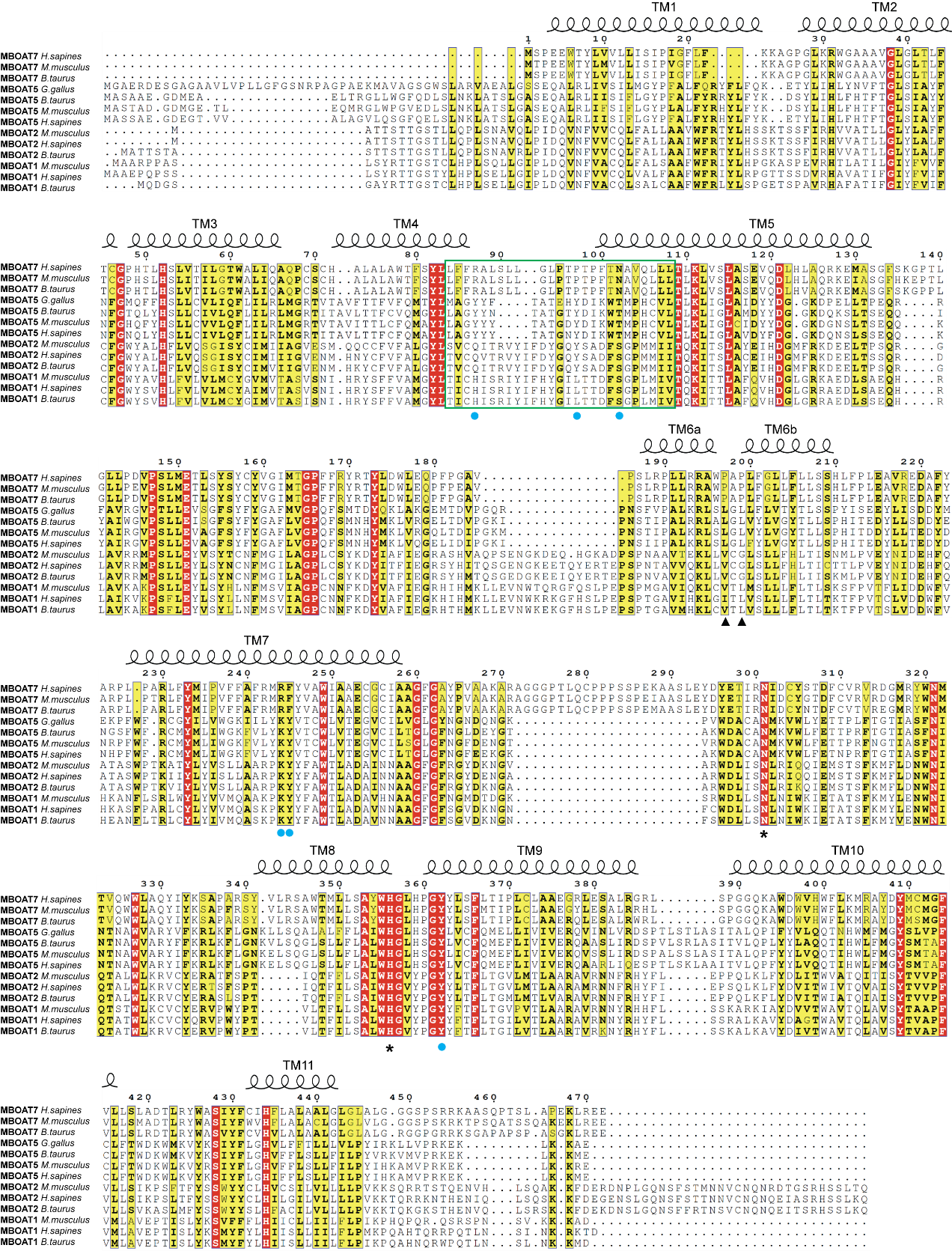


**Supplementary Fig. 8 | Sequence alignments of MBOAT1, 2, 5 and 7.** The sequences were aligned using the Clustal Omega Server. Secondary structural elements of human MBOAT7 are marked above the alignment. Residues were colored based on their conservation using the ESPript server. Catalytic residues are denoted by asterisks. Two conserved proline residues of MBOAT7 were denoted by solid triangles. Important residues that interact with the inositol groups are denoted by solid cyan dots. The variable regions that determine the substrate specificity is highlighted by a green frame.


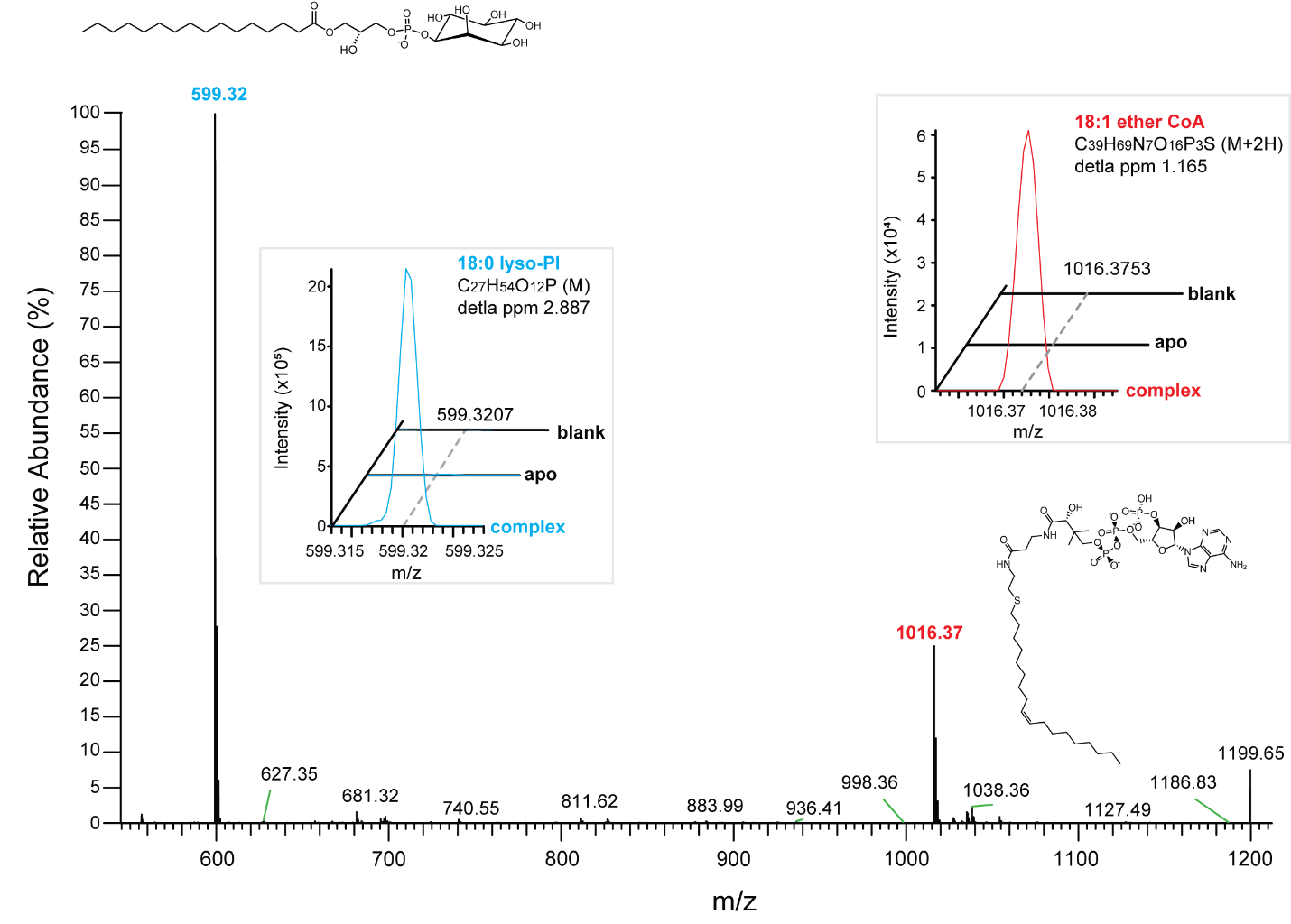


**Supplementary Fig. 9 | Mass spectrometry analysis of the substrate incorporation into the complex protein preparation.** The main panel shows the spectrum of the standards of two substrates. Their m/z values and molecular structures are shown as well. Two inserts show the quantification of individual substrates, 18:0 lyso-PI (cyan) and 18:1 ether CoA (red) in blank, apo-protein preparation and the complex protein prep.


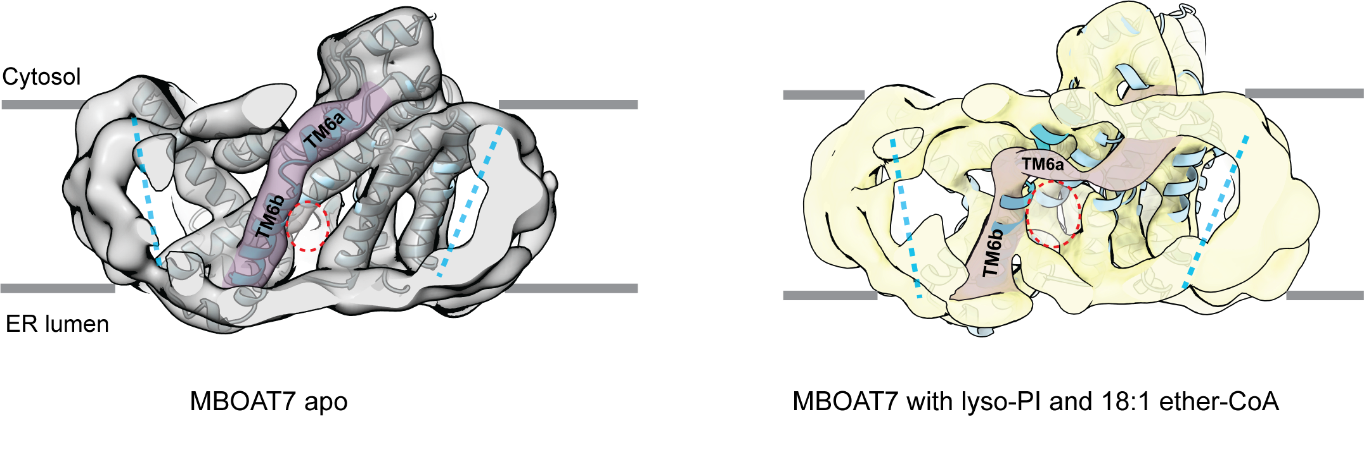


**Supplementary Fig. 10 | Comparison of MBOAT7 apo-structure and MBOAT7 structure with lyso-PI and 18:1 ether-CoA.** The conformational change of TM6 is highlighted in purple. MBOAT7 apo map is low pass filtered to 6 Å, and both maps are contoured at 6 σ. MBOAT7 apo model is docked into both maps. The lateral channel entrances are approximately indicated by red circles with dashed lines.


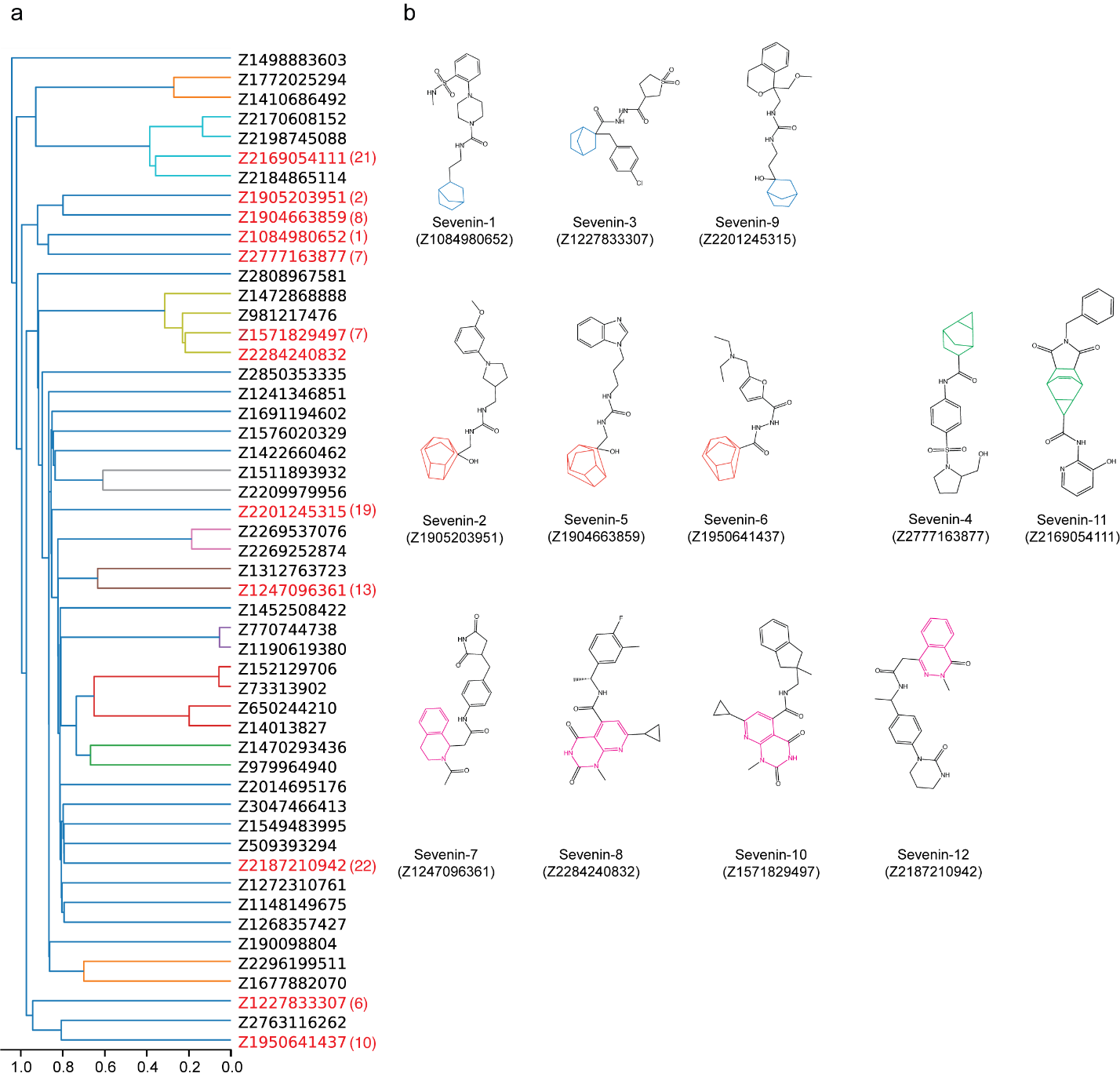


**Supplementary Fig. 11 | Similarity analysis of the top VirtualFlow candidates. a**, Hierarchical clustering of fingerprints from the top 50 VirtualFlow candidates selected based on their free-energy scores. The synthesized compounds are highlighted in red, and the numbers in parentheses indicate the ranking of the compounds based on free-energy scores. **b,** Classification of the 12 synthesized compounds based on their signature substructures, which are highlighted in different colors.


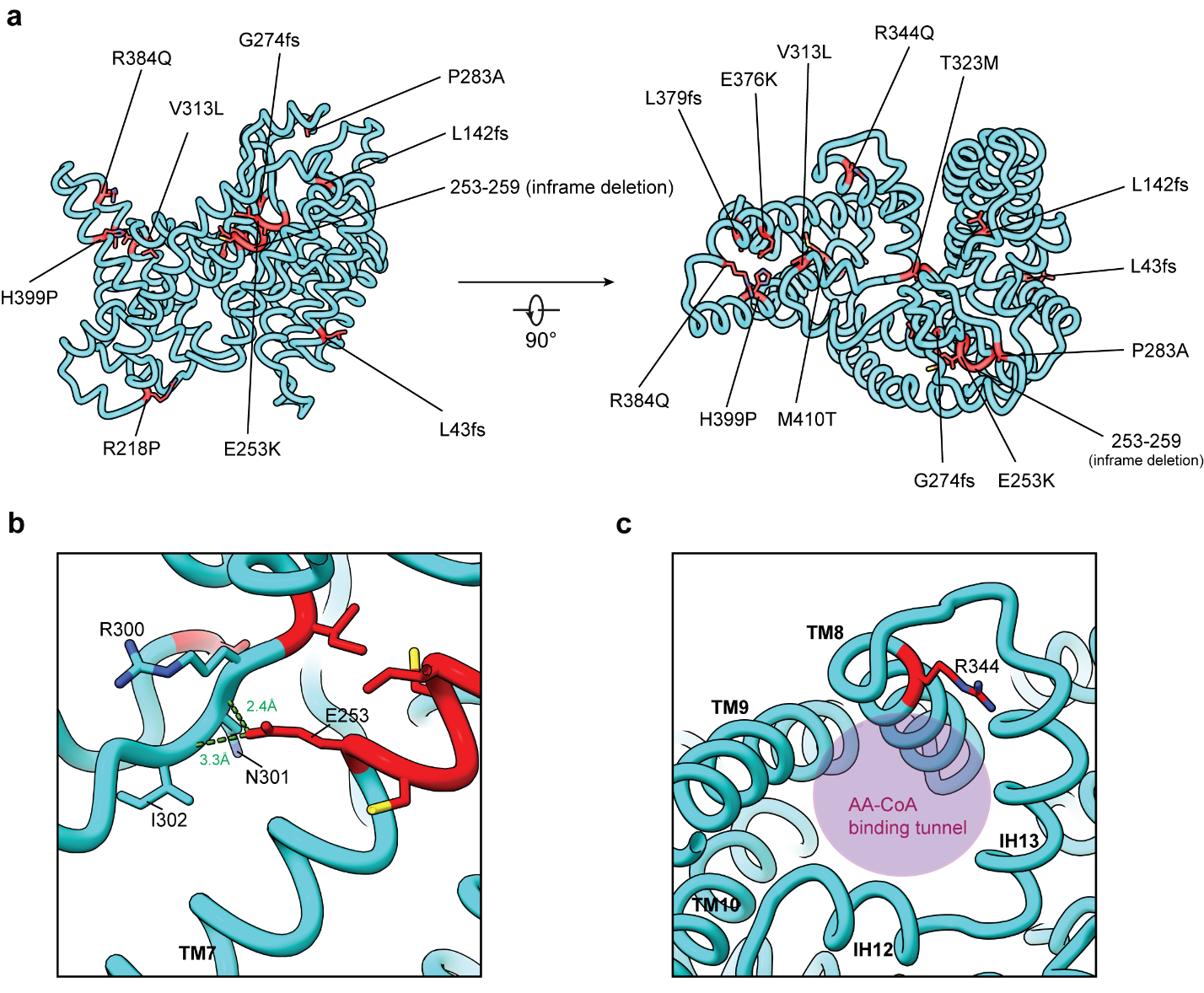


**Supplementary Fig. 12 | Mapping disease-causing mutations to the MBOAT7 structure. a**, The disease-causing mutations are highlighted in red with side chains. These mutations are correlated with forms of intellectual developmental disorders. fs, frame shift. **b**, Interaction between E253 and the backbone Ns of N301 and I302. Hydrogen bonds are shown in green dashed lines with indicated bond lengths. **c**, R344 is located near the entrance of the acyl-CoA binding tunnel. The arachidonyl-CoA (AA-CoA) binding tunnel is highlighted with a pink circle.

**Supplementary Table 1 | Cryo-EM data collection, refinement and validation statistics**

|  | MBOAT7 in PMAL-C8  (EMD-28552)  (PDB 8ERC) | |
| --- | --- | --- |
| **Data collection and processing** |  |  |
| Magnification | 105,000 |  |
| Voltage (kV) | 300 |  |
| Electron exposure (e–/Å^2^) | 50 |  |
| Defocus range (μm) | 0.8 – 2.2 |  |
| Pixel size (Å) | 0.825 |  |
| Symmetry imposed | C1 |  |
| Initial particle images (no.) | 25,824,808 |  |
| Final particle images (no.) | 206,418 |  |
| Map resolution (Å)  FSC threshold | 3.7  0.143 |  |
| Map resolution range (Å) | 2.8 – 10.9 |  |
|  |  |  |
| **Refinement** |  |  |
| Initial model used | AF-Q96N66-F1-model_v2 (Alphafold2 model) |  |
| Model resolution (Å)  FSC threshold | 3.18  0.143 |  |
| Model resolution range (Å) | 211.2 - 3.0 |  |
| Map sharpening *B* factor (Å^2^)  3D FSC sphericity score | -120  0.87 |  |
| Model composition  Non-hydrogen atoms  Protein residues | 3510  441 |  |
| *B* factors (Å^2^)  Protein | 105 |  |
| R.m.s. deviations  Bond lengths (Å)  Bond angles (°) | 0.003  0.671 |  |
| Validation  MolProbity score  Clashscore  Poor rotamers (%) | 2.1  8.11  0 |  |
| Ramachandran plot  Favored (%)  Allowed (%)  Disallowed (%)  Map-model fit  Atom inclusion  Q-score | 97.72  2.28  0  0.4849 (contour level=5.5)  0.361 (contour level=5.5) |  |
