# Supplementary material for: The structure of phosphatidylinositol remodeling MBOAT7 reveals its catalytic mechanism and enables inhibitor identification
Source: Nat Commun. 2023 Jun 14;14:3533. doi: 10.1038/s41467-023-38932-5 (PMC10267149; doi:10.1038/s41467-023-38932-5)
Supplement: Supplementary file 3 — Description of Additional Supplementary Files [file 41467_2023_38932_MOESM3_ESM.pdf]

Description of Additional Supplementary Files

Supplementary Data 1: the primers used in this study.
